# Artificial Neural Network to Modeling Zero-inflated Count Data: Application to Predicting Number of Return to Blood Donation

**Published:** 2017-09-02

**Authors:** Shima Haghani, Morteza Sedehi, Soleiman Kheiri

**Affiliations:** ^1^ Department of Biostatistics and Epidemiology, Faculty of Public Health, Shahrekord University of Medical Sciences, Shahrekord, Iran

**Keywords:** Blood donation, Count regression Models, Zero-inflated count data, Artificial neural network

## Abstract

**Background:** Traditional statistical models often are based on certain presuppositions and limitations that may not presence in actual data and lead to turbulence in estimation or prediction. In these situations, artificial neural networks (ANNs) could be suitable alternative rather than classical statistical methods.

**Study design:** A prospective cohort study.

**Methods:** The study was conducted in Shahrekord Blood Transfusion Center, Shahrekord, central Iran, on blood donors from 2008-2009. The accuracy of the proposed model to prediction of number of return to blood donations was compared with classical statistical models. A number of 864 donors who had a first-time successful donation were followed for five years. Number of return for blood donation was considered as response variable. Poisson regression (PR), negative binomial regression (NBR), zero-inflated Poisson regression (ZIPR) and zero-inflated negative binomial regression (ZINBR) as well as ANN model were fitted to data. MSE criterion was used to compare models. To fitting the models, STATISTICA 10 and, R 3.2.2 was used

**Results:** The MSE of PR, NBR, ZIPR, ZINBR and ANN models was obtained 2.71, 1.01, 1.54, 0.094 and 0.056 for the training and 4.05, 9.89, 3.99, 2.53 and 0.27 for the test data, respectively.

**Conclusions:** The ANN model had the least MSE in both training, and test data set and has a better performance than classic models. ANN could be a suitable alternative for modeling such data because of fewer restrictions.

## Introduction

In regression models, when outcome is a count variable, Poisson Regression (PR) or negative binomial regression (NBR) is used for modeling^1^. Poisson distribution is used if the mean and variance of the data on response variable are equal and negative binomial distribution is suitable if the variance is larger than the mean (count variable is largely dispersed) ^2, 3^. In real situations, during modeling count outcomes, we frequently face two issues namely overdispersion and excess zeroes in outcome values. Because of excess zeroes in response variable, mean and variance of response variable are not equal. Therefore, Poisson is not a suitable model for this type of data. In these specific situations, models such as zero-inflated Poisson regression (ZIPR), zero-inflated negative binomial regression (ZINBR), hurdle model, and generalized Poisson model have been recommended^4-7^.

Generally, classical statistical models have some presuppositions and limitations, such as equal variances of errors, considering a default distribution for the response variables, and linear relationship between dependent as well as independent variables that in actual data may not be available. In addition, most of these approaches have not the capability of modeling sophisticated, non-linear relationships and high degree interactions. Sensitivity to missing values and outliers is another limitation of these models^8^.

A potential approach that able to overcome the limitations of classical models could be artificial neural networks (ANNs). Multi-layer perceptron (MLP) is the most popular architecture in ANNs. Usually, back-propagation (BP) algorithm is used to learn MLP based on minimizing sum of squared errors^9^. Generalizability of ANNs allows the model to provide an appropriate answer related to a new observation. Since the precise and accurate prediction is very important in medicine, so, using models with highest confidence is a priority and ANN model seems to be a suitable method for this purpose^8^.

Blood, as a mysterious liquid, is part of the body’s vital system with special characteristics enabling it to save life of a patient or an individual in need through being donated. This issue is more important than one might think, as one per three individuals' needs transfusion of blood and its products^10^.

Despite all advances made in different medical fields, no artificial substitute has been yet found for blood to satisfy the needs of different patients and the only route to meeting the need for this vital substance is the blood donated or bought^10^.

A human can donate blood several times during lifetime. Donors who donate blood at least once per six months are classified as constant donors. The number of blood donations by these donors is definite and their blood health is certain, therefore it is more suitable for health system to enhance index of constancy as much as possible. Women can donate blood at most three times per year and men can do it once per three months^11, 12^. Therefore, predicting the number of blood donations has a particular status and hence we should seek for an appropriate, highly accurate approach to predicting this number.

In this study, we proposed a new method with fewer restrictions based on ANN to model zero-inflated count responses, then, the accuracy of the proposed model was compared with common statistical and zero-inflated models to prediction of a number of returns to blood donations.

## Methods

**Figure 1:**
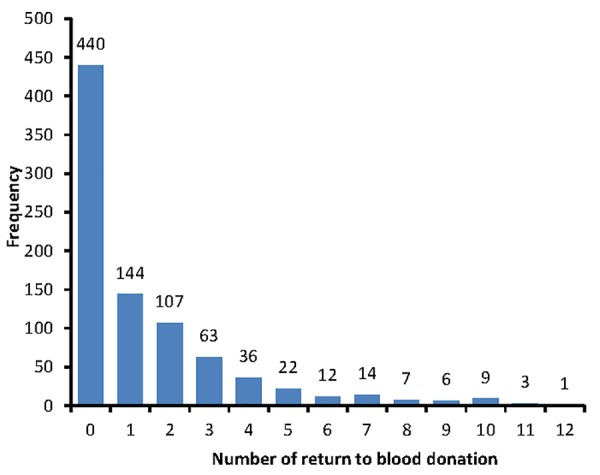
Frequency distribution of the number of return to blood donations

To compare ANN with classic models data from a longitudinal study was used. The study was designed as a follow-up study with a maximum of five years conducted in Shahrekord Blood Transfusion Center, Shahrekord, central Iran. At the beginning of the study, a list of registered donors in Negareh software system used by the Shahrekord Blood Transfusion Center who had blood donations for the first time from 21 Mar 2008 until 20 Mar 2009 was prepared. The sampling method was systematic sampling and the sample size was calculated using previous information about percentage of donors return to blood donation for at least five times^10^. The number of return to blood donation until 20 Mar 2013 were extracted as response variable and sex, age, weight, marital status, education, job, blood group and Rh were considered as independent variables. Figure 1 shows the frequency of number of return to blood donation. Overall, 440 numbers (50.9%) of return to blood donation was zero. Therefore, zero-inflated models should be used for modeling data. For fitting models, 70% cases were used as training set, and 30% were used as test set.

As discussed above, for count regression, models in the case of excess of zeroes in response variable, Poisson, and negative binomial models are inadequate and zero-inflated model is alternative way to model data^7^. The probability density function for zero-inflated cunt data can be formulated as follows:

**Figure:**
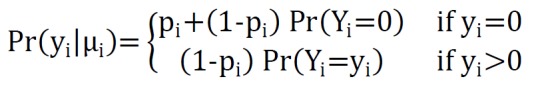


That p_i_ is proportion of extra zeroes than original Poisson or negative binomial in response variable and Pr(Y_i_=y_i_) is probability of Y_i_=y_i_ in Poisson or negative binomial distribution. By replacing μβ^T^x_i_=e^ β^T^x_i_^ in above formula, ZIPR model defined as:

**Figure:**
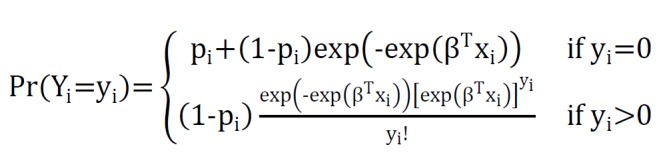


And ZINBR model can be written as:

**Figure:**
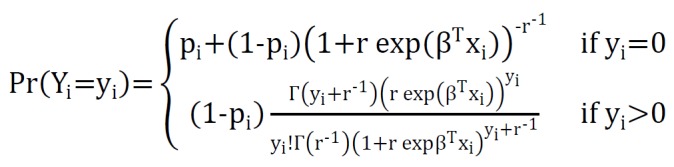


That r is overdispersion parameter^5^.

For fitting of ANN model, MLP with one hidden layer was used. ANN adopts a set of input observations, x_i_, and compute outputs y_i_, using a specified number of layers. The architecture of ANN model can be written as:

$$y_{i}=\psi_{o}(\beta_{0}+\sum_{j=1}^{M}\beta_{j}\psi_{h}(w_{j0}+\sum_{s=1}^{P}x_{is}w_{js}))i=1,...,n$$

where w_js_ is the weight for input x_is_ at the hidden node j. In addition, β_j_ is the weight dependent to the hidden node j, and w_j0_ and β_0_ are the biases for the hidden and the output nodes respectively. In addition, p and M are number of covariates and number of nodes in hidden layer respectively. The function Ψ_h_ is activation functions of hidden layer and the function Ψ_o_ is activation functions of output layer^8^.

The BP algorithm was used to learning MLP based on minimizing sum of squared errors. The BP algorithm has two computational paths; Forward path and backward path. For the k-th input, the equations on the forward path were as follows:

**Figure:**
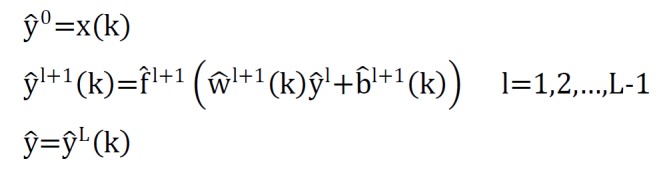


In forward path, the network parameters do not change during computing, and the activation functions applied on each neuron:

**Figure:**
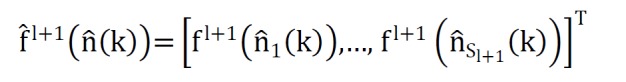


In backward path, the sensitivity matrices from the last layer were returned to the first layer:

**Figure:**
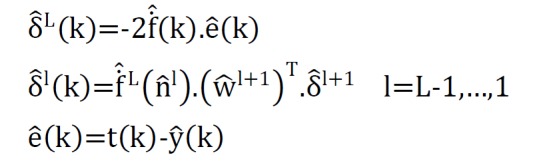


Finally, the weights and biases matrix were regulated by the following relationships:

**Figure:**
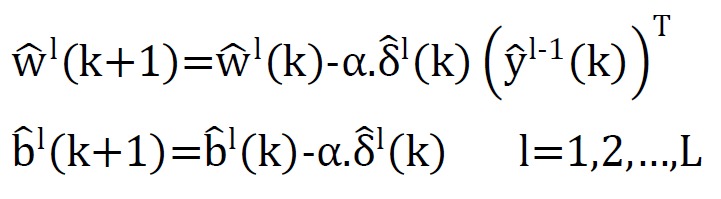


In recent formulas, L, f, nl , α, δl, t(k) and ek were referred to number of network layers, activation function, output in hidden layer, network learning rate, transformation gradient in l-th layer, the actual output value of k-th sample and Estimated error for k-th sample respectively^12^.

Accuracy of statistical count regression models, including PR, NBR, ZIPR, and ZINBR were compared with ANN model to prediction number of return to blood donation via MSE criterion. We fitted MLP with one hidden layer, including 11-18 nodes. Batch Gradient Descent (BGD), Conjugate Gradient (CG) and Broyden Fletcher Goldfarb Shanno (BFGS) learning algorithms were used for training. All these algorithms are from BP algorithm family^13^. To fitting the models, STATISTICA 10 and, R 3.0.1 was used.

## Results

From 864 donors, 801(92.7%) donors were male and 623(72.1%) were married. Overall, 710(82.2%) of donors lived in the city and 154(17.8%) in the country. Mean ± standard deviation of age in donors at the first donation was 36.6±10.7 yr and the mean of body weight at the first donation was 77.8±11.7 kg. Number of return to blood donation was from 0 to 12 (Figure 1). Mean and standard deviation of number of return to blood donations was 1.41 and 2.16 respectively. Overall, 440 (50.9%) of donors did not return to donate blood. The frequency of successful return to blood donation with respect to blood type, donors' Rh, marital status, stay, education level and job class is shown in Table 1.

**Table 1:** Frequency of donation based on general characteristic of donors

**Variables**	**Return to blood donation, n=440**		**Not return to blood donation, n=424**	
	**Number**	**Percent**	**Number**	**Percent**
Blood type				
AB	32	55.2	26	44.8
B	83	52.2	76	47.8
A	150	53.0	133	47.0
O	175	48.1	189	51.9
Rh				
Positive	403	50.8	390	49.2
Negative	37	52.1	34	47.9
Marital status			
Married	317	50.9	306	49.1
Single	123	51.0	118	49.0
Stay				
Urban	366	51.5	344	48.5
Rural	74	48.1	80	51.9
Education				
Elementary	89	56.3	69	43.7
High School	105	50.7	102	49.3
Diploma	160	52.5	145	47.5
University	86	44.3	108	55.7
Job group				
Housekeeper	38	74.5	13	25.5
Clerical	67	39.9	101	60.1
Worker	71	54.6	59	45.4
Free Job	194	50.3	192	49.7
Student	70	54.3	59	45.7

For Akaike information criterion (AIC), NBR and ZINBR models have a similar performance, but MSE criterion is 0.96 for NBR and 1.03 for ZINBR (Table 2).

**Table 2:** Comparison statistical models in all data

**Model**	**PR**	**NBR**	**ZIPR**	**ZINBR**
**AIC**	3376.51	2745.82	2895.74	2744.96
**MSE**	2.58	1.037	1.45	0.97

PR: Poisson Regression, NBP: Negative Binomial Regression, **ZIPR:** Zero-Inflated Poisson Regression,** ZINBR:** Zero-Inflated Negative Binomial Regression

To find the best structure of ANN model, CG, BGD and BFGS training algorithms were compared and BFGS selected (Table 3). For BFGS algorithm, 4-8 neurons and four different activation functions in hidden layer with hyperbolic tangent activation function in the external layer were developed and compared (Table 4). Finally, regression and ANN models were compared with MSE criterion (Table 5).

**Table 3:** MSE of training ANN algorithms in training and test data set

**Training Algorithm**	**BGD**	**CG**	**BFGS**
Training set	0.186	0.063	0.072
Test set	0.226	0.562	0.218

**BGD:** Batch Gradient Descent, **CG:** Conjugate Gradient, **BFGS:** Broyden Fletcher Goldfarb Shanno.

**Table 4:** MSE of different activation functions and number of nodes in middle layer for BFGS algorithm

**No. of nodes in middle layer**	**Linear**	**Logistic**	**Exponential**	**Hyperbolic tangent**
11	0.1904	0.1066	0.1327	0.1004
12	0.1904	0.0954	0.1224	0.0956
13	0.1904	0.0925	0.1223	0.0795
14	0.1904	0.0813	0.1101	0.0814
15	0.1904	0.0761	0.1162	0.0730
16	0.1904	0.0849	0.1119	0.0612
17	0.1904	0.0717	0.1020	0.0563
18	0.1904	0.0734	0.1042	0.0684

**Table 5:** MSE of ANN and statistical models

**Model**	**ANN**	**PR**	**ZIPR**	**NBR**	**ZINBR**
Training set	0.05	2.71	1.54	1.01	0.09
Test set	0.27	4.05	3.99	9.89	2.53

**ANN:** Artificial Neural Network,** PR:** Poisson Regression, **NBP:** Negative Binomial Regression**, ZIPR:** Zero-Inflated Poisson Regression,** ZINBR:** Zero-Inflated Negative Binomial Regression

## Discussion

Healthy blood supply is one of the most important issues in blood transfusion organizations around the world. On the other hand, it is very important to recognize who is able to donate healthy blood continuously. Therefore, providing models that can accurately predict the number of return to blood donation(s) is very valuable.

In this study, response variable was the number of return to blood donation that was a zero-inflated count variable. To model such variables, the use of common methods for analyzing count data in classical statistics will be associated with errors. It is very important to know the methods predicted with high precision.

In this study, we presented different approaches for modeling zero-inflated count outcomes. ANN and count statistical models such as PR, NBR, ZIPR, and ZINBR were compared and more accurate model for predicting the number of return to donations was determined. ZINBR was the best model among classical statistical models to predicting a number of return to blood donation, while, comparison between these approaches and ANN in view of MSE values indicated that ANN could be a more appropriate approach to prediction. In a longitudinal study investigating the performance of Poisson regression of neural networks in predicting main cognitive changes compared artificial neural network and Poisson regression for cognitive changes in the elderly within a five-year follow-up through MSE, ANN with any structure had a better performance than Poisson regression^14^.

NBR and ANN analyzed the frequency of accidents in freeways in some highways in Taiwan. Artificial neural network had better performance compared with statistical models for prediction^15^.

In zero-inflated count data, the common methods in classic statistics suffer from some shortcomings. Their performances depend on the distribution of variables, size, and quality of data, etc. When relation between predictors and response variable is nonlinear, predictions will be confusing and will lack confidence. ANN can be considered as alternative techniques to overcome this problems^8^.

Despite the advantages of ANN models, they have some limitations. They are neither capable to inference on the parameters nor to assess significance of relationship between the variables^8^.

## Conclusion

ANN model had the best performance of prediction of number of return to blood donation in both training and test data set compared with PR, NBR, ZIPR and ZINBR models. Therefore, considering the importance of precise prediction in medical studies and due to the restrictions of traditional statistical methods, the use of ANN model is a suitable alternative for analyzing such data.

## Conflict of interest statement

The authors declare that there is no conflict of interest.

## Funding

The present study was extracted from MSc thesis supported by a grant number 1632 from the Research and Technology Deputy of Shahrekord University of Medical Sciences.

## Highlights

- ZINBR was the best model among classical statistical models to predicting number of return to blood donation
- The ANN model had the least MSE in both training and test data set
- ANN model had the best performance of prediction of number of return to blood donation in both training and test data set compared with PR, NBR, ZIPR and ZINBR models
